# Supplementary material for: The Systems Biology Research Tool: evolvable open-source software
Source: BMC Syst Biol. 2008 Jun 29;2:55. doi: 10.1186/1752-0509-2-55 (PMC2446383; doi:10.1186/1752-0509-2-55)
Supplement: Additional file 1 — SBRT Archive. An archive of the current version of the Systems Biology Research Tool. [file 1752-0509-2-55-S1.zip › sbrt-1.4.0/doc/developers_guide/api/index-files/index-23.html]

W-Index


|  |  |  |
| --- | --- | --- |
| **Overview** \| Package \| Class \| Use \| **Tree** \| **Deprecated** \| **Index** \| **Help** | |  |
| **PREV LETTER**   **NEXT LETTER** | **FRAMES**    **NO FRAMES**     **All Classes** |  |


A B C D E F G H I J K L M N O P Q R S T U V W X Y Z 

---


## **W**

**warn(String)** - Method in interface sbrt.kernel.io.WarningHandler: Processes the provided warning message in an appropriate way. **WarningHandler** - Interface in sbrt.kernel.io: This interface is used to represent a class of objects that deal with warning messages. **WarningHandlerFactory** - Class in sbrt.kernel.io: This class is used to provide a default `WarningHandler`. **WarningHandlerFactory()** - Constructor for class sbrt.kernel.io.WarningHandlerFactory: **WhitespaceListFormat**<E> - Class in sbrt.shell.text: This class is used to define a format for whitespace-delimited lists. **WhitespaceListFormat(SimpleFormat<E>)** - Constructor for class sbrt.shell.text.WhitespaceListFormat: Constructs a new whitespace-delimited list format with the provided element format. **WhitespaceListFormat(Formatter<E>, Parser<E>)** - Constructor for class sbrt.shell.text.WhitespaceListFormat: Constructs a new whitespace-delimited list format with the provided element format. **WhitespaceSetFormat**<E> - Class in sbrt.shell.text: This class is used to define a format for whitespace-delimited sets. **WhitespaceSetFormat(SimpleFormat<E>)** - Constructor for class sbrt.shell.text.WhitespaceSetFormat: Constructs a new whitespace-delimited set format with the provided element format. **WhitespaceSetFormat(Formatter<E>, Parser<E>)** - Constructor for class sbrt.shell.text.WhitespaceSetFormat: Constructs a new whitespace-delimited set format with the provided element format. **widthFormat(String, int)** - Static method in class sbrt.kernel.util.Util: Attemps to format the provided string such that system-dependent newline characters appear no more than `maxWidth` characters apart. **WorkingDirectory** - Class in sbrt.shell.io: This class is used to manage the working directory of the SBRT. **WorkingDirectory()** - Constructor for class sbrt.shell.io.WorkingDirectory: **write(int)** - Method in class sbrt.shell.gui.TextAreaOutputStream: Writes the specified byte as a character to the javax.swing.JTextArea. **write(int)** - Method in class sbrt.shell.gui.TextPaneOutputStream: Writes the specified byte as a character to the javax.swing.JTextArea. **write(IrrevRxnFluxome, String)** - Method in class sbrt.shell.proc.fba.SbmlWriter: Writes the provided fluxome in SBML format to a file with the provided name. **writeFile(IrrevRxnFluxome, OutputFile<String>)** - Method in class sbrt.shell.proc.fba.MetatoolFileWriter: Writes a metatool input file. **writeFluxBounds(String)** - Method in class sbrt.shell.proc.fba.BiggSbmlReader: Writes the flux bounds contained in the SBML file to a file with the provided name. **writeFluxBounds(String)** - Method in class sbrt.shell.proc.fba.PalssonSbmlReader: Writes the flux bounds contained in the SBML file to a file with the provided name. **writeFluxBounds(String)** - Method in class sbrt.shell.proc.graph\_theory.RedundantCycleRemoval: Writes the flux bounds contained in the SBML file to a file with the provided name. **writeFluxome(IrrevRxnFluxome, String)** - Static method in class sbrt.shell.mng.fba.FbaManager: Writes the provided fluxome to a file with the provided name. **writeFluxome(IrrevRxnFluxome, String, PrintWriter)** - Static method in class sbrt.shell.mng.fba.FbaManager: Writes the provided fluxome to a file with the provided name. **writeFluxVector(String)** - Method in class sbrt.shell.proc.fba.BiggSbmlReader: Writes the flux vector contained in the SBML file to a file with the provided name. **writeFluxVector(String)** - Method in class sbrt.shell.proc.fba.PalssonSbmlReader: Writes the flux vector contained in the SBML file to a file with the provided name. **writeFluxVector(String)** - Method in class sbrt.shell.proc.graph\_theory.RedundantCycleRemoval: Writes the flux vector contained in the SBML file to a file with the provided name. **writeGenes(String)** - Method in class sbrt.shell.proc.fba.BiggSbmlReader: Writes the genes contained in the SBML file to a file with the provided name. **writeGenes(String)** - Method in class sbrt.shell.proc.fba.PalssonSbmlReader: Writes the genes contained in the SBML file to a file with the provided name. **writeGenes(String)** - Method in class sbrt.shell.proc.graph\_theory.RedundantCycleRemoval: Writes the genes contained in the SBML file to a file with the provided name. **writeIntersection(String, Map<String, Double>, Map<String, Double>, PrintWriter)** - Static method in class sbrt.shell.mng.stat.CorrelationManager: Writes the intersection of the provided vectors to a file with the specified name. **writeLine(String)** - Method in class sbrt.shell.io.AbstractMapOutputFile: Writes the provided string to this file as a line. **writeLine(String)** - Method in class sbrt.shell.io.TextOutputFile: Writes the provided string to this output file. **writeObject(Object)** - Method in class sbrt.shell.io.SerializedOutputFile: Serializes the provided object and writes it to this file. **writeProblem(String)** - Method in class sbrt.ext.cplex.CplexSolver: Writes the CPLEX model to a file with the provided name. **writeProteins(String)** - Method in class sbrt.shell.proc.fba.PalssonSbmlReader: Writes the proteins contained in the SBML file to a file with the provided name. **writeRxns(String)** - Method in class sbrt.shell.proc.fba.BiggSbmlReader: Writes the reactions contained in the SBML file to a file with the provided name. **writeRxns(String)** - Method in class sbrt.shell.proc.fba.PalssonSbmlReader: Writes the reactions contained in the SBML file to a file with the provided name. **writeRxns(String)** - Method in class sbrt.shell.proc.graph\_theory.RedundantCycleRemoval: Writes the reactions contained in the SBML file to a file with the provided name. **WwFluxomeReducer** - Class in sbrt.kernel.fba.comp: This class is used to reduce the size of fluxomes for the purpose of identifying the steady state cycles (Type III extreme pathways) they contain. **WwFluxomeReducer(ProgramSolver<Object, Object>)** - Constructor for class sbrt.kernel.fba.comp.WwFluxomeReducer: Constructs a fluxome reducer from the provided linear program solver. **WwFluxomeReducerManager** - Class in sbrt.shell.mng.fba: This class is used to manage the WW fluxome reduction algorithm. **WwFluxomeReducerManager()** - Constructor for class sbrt.shell.mng.fba.WwFluxomeReducerManager

---


|  |  |  |
| --- | --- | --- |
| **Overview** \| Package \| Class \| Use \| **Tree** \| **Deprecated** \| **Index** \| **Help** | |  |
| **PREV LETTER**   **NEXT LETTER** | **FRAMES**    **NO FRAMES**     **All Classes** |  |


A B C D E F G H I J K L M N O P Q R S T U V W X Y Z 

---
